# Caveolin-1 Regulates Rac1 Activation and Rat Pulmonary Microvascular Endothelial Hyperpermeability Induced by TNF-α

**DOI:** 10.1371/journal.pone.0055213

**Published:** 2013-01-30

**Authors:** Min Shao, Yang Yue, Geng-Yun Sun, Qing-Hai You, Nan Wang, Dan Zhang

**Affiliations:** 1 Department of Respiratory Medicine, The First Affiliated Hospital of Anhui Medical University, Hefei, Anhui, People’s Republic of China; 2 Department of Critical Care Medicine, Anhui Provincial Hospital Affiliated to Anhui Medical University, Hefei, Anhui, People’s Republic of China; National Institutes of Health, United States of America

## Abstract

A multiplicity of vital cellular and tissue level functions are controlled by caveolin-1 and it is considered to be an important candidate for targeted therapeutics. Rac1-cortactin signaling plays an important role in maintaining the functions of the endothelial barrier in microvascular endothelial cells. The activity of Rac1 has been shown to be regulated by caveolin-1. Therefore, the present study investigated the consequences of down-regulating caveolin-1 and the subsequent changes in activity of Rac1 and the endothelial barrier functions in primary rat pulmonary microvascular endothelial cells (RPMVECs). RPMVECs were transfected with a small hairpin RNA duplex to down-regulate caveolin-1 expression. This procedure significantly increased the activity of Rac1. Moreover, down-regulation of caveolin-1 attenuated TNF-α-induced decrease in TER, increase in the flux of FITC-BSA and the disappearance of cortactin from the cell periphery in RPMVEC. Rac1 inhibitors significantly abolished this barrier-protective effect induced by down-regulation of caveolin-1 in response to TNF-α in RPMVECs. In conclusion, our data suggest a mechanism for the regulation of Rac1 activity by caveolin-1, with consequences for activation of endothelial cells in response to TNF-α.

## Introduction

Caveolae or “small caves” were originally identified as 50 to 100 nm flask-shaped, non-clathrin-coated invaginations of the plasma membrane. Recent data revealed the implication of caveolae in a variety of cellular processes, including transendothelial vesicular transport [1], [2], internalization of pathogens [3], integration of signaling pathways [4], and regulation of cell proliferation [5]. Caveolae are stabilized by caveolins that include three groups (caveolin-1, -2, and -3). In endothelial cells (ECs), caveolae contain caveolin-1 and caveolin-2. Caveolin-1 is considered to be an important protein, which contains a scaffolding domain, involved in the localization and regulation of several signaling cascades [6]. Caveolin-1 also acts as an inhibitory regulator of endothelial Rac1 signaling at the caveolae membrane [7]. The ability of caveolin-1(Cav-1) to regulate Rac1 signaling suggests a possible function of caveolin-1 involved in regulating endothelial permeability. Although the caveolin-1-Rac1 interaction has been implicated in the cellular processes of vascular hypertrophy [8] and cell migration [9], there is no evidence regarding the role of caveolin-1 in regulating Rac1 signaling in TNF-α-induced hyperpermeability of pulmonary microvascular endothelial cells.

Previous studies have proposed and envisaged a working model of EC barrier regulation [10], [11]. According to this model, the formation of paracellular gaps is regulated by the equilibrium between the forces providing centripetal tension and forces ensuring cell spreading and opposing cell collapse. Whereas centripetal tension is imposed by the actomyosin cytoskeleton, centrifugal forces are imposed by focal adhesions and adheren junctions. These elements (focal adhesions and adherens junctions) are anchored to the underlying cortical actin ring, which serves to fortify the cell periphery. The attachment of cortical rings to the membrane and its dynamic rearrangement is modulated by an array of actin and/or membrane-binding proteins. The equilibrium between the centripetal and centrifugal forces is a subject to regulation by several signaling pathways [10], [12].

The Rho GTPase family [13], especially RhoA, Rac1, and Cdc42, control the dynamic and structure of F-actin filaments that determines cell shape, facilitates cell adhesion to the sub-endothelial matrix, and participates in the regulation of junctional complexes [14]. The balance between RhoA and Rac1-mediated signaling may be a key point of EC barrier regulation. Previous reports have identified that Rac1 is interrelated to the maintenance and stabilization of microvascular endothelial barrier functions, whereas RhoA drives endothelial barrier instability [15]. Rac1 GTPases act as molecular switches, cycling between the GTP-bound ‘‘active’’ form, and the GDP-bound ‘‘inactive’’ form [16]. In addition, activation of Rac1 plays a role in the maintenance of barrier integrity under a resting state and appears to be a possible approach to protect barrier functions via strengthening the cortical actin cytoskeleton to enhance the stiffness of the cell periphery under inflammatory conditions [17].

The cortical actin band spans the entire circumference of endothelial cells and it is composed of F-actin bundles. Cortactin, an actin-binding protein, is a ubiquitously expressed tyrosine kinase and has been implicated in cortical actin assembly and reorganization. Indeed, the functional relevance of cortactin for endothelial permeability was demonstrated by attenuated responses to barrier-protective stimuli following cortactin knockdown [18]. Previous studies have reported that Rac1 can strengthen the cortical actin cytoskeleton by promoting cortactin to accumulate at the cell border and this may be effective in enhancing endothelial barrier properties.

In all, considered the important role of Rac1 in controlling cell permeability and the close relationship between Rac1 and caveolin-1, we hypothesized that it was reasonable to explore whether caveolin-1 is involved in regulating endothelial permeability induced by TNF-α through the Rac1 signaling pathway. Our current study provides evidence that TNF-α-induced endothelial barrier breakdown occurs by impairing Rac1 signaling and this process requires caveolin-1 participation. We also found that primary rat pulmonary microvascular endothelial cells (RPMVECs) lacking caveolin-1 were significantly resistant to TNF-α-induced barrier dysfunction by up-regulating Rac1 activity. Therefore, this study describes a novel mechanism by which the down-regulation of caveolin-1 confers cytoprotection to RPMVECs in response to TNF-α.

## Materials and Methods

### Reagents

TNF-α was purchased from Peprotech Inc. (Rocky Hill, NJ). Caveolin-1 antibody was purchased from Abcam Inc. (Cambridge, MA). Polyclonal anti-cortactin antibody, rhodamine-conjugated phalloidin, FITC-labeled BSA and unlabeled BSA were purchased from Sigma-Aldrich (St. Louis, MO). NSC-23766 was obtained from Calbiochem/Merck (Darmstadt, Germany). 8-pCPT-20 -O-Methyl-cAMP (O-Me-cAMP) was from Biolog Life Science Institute (Bremen, Germany). Horseradish peroxidase (HRP)-labeled antibody to rabbit IgG was purchased from Wuhan Boster Biological Engineering Co, Ltd (Wuhan, China). Transwell clear polyester membrane cell culture chambers (24-well type, 6.5 mm diameter, 0.4 µm pore size) were purchased from Costar (Cambridge, MA). High glucose Dulbecco’s modified Eagle’s medium (DMEM) were purchased from Invitrogen (Carlsbad, CA). Fetal calf serum, penicillin, streptomycin, and trypsin were purchased from HyClone Laboratory (Salt Lake City, UT).

### Primary RPMVECs Culture

As described previously, the method of isolation and culture primary RPMVECs has been successfully established in our laboratory [19], [20]. Briefly, Wistar rats were maintained under specific pathogen-free and controlled light conditions (22°C, 55% humidity, and 12-hour day/night rhythm). Primary rat pulmonary microvascular endothelial cells (RPMVECs) were grown in a humidified atmosphere with 5% CO_2_. Experimental data were obtained from cells in their third to fifth generation. This study was carried out in strict accordance with the recommendations in the Guide for the Care and Use of Laboratory Animals of the National Institutes of Health. The protocol was approved by the Committee on the Ethics of Animal Experiments of Anhui Medical University. All surgeries were performed under sodium pentobarbital anesthesia, and all efforts were made to minimize suffering.

### Depletion of Caveolin-1 in RPMVEC

Caveolin-1 RNAi lentiviruses were packaged with the use of a four-plasmid system as described previously [21]. Two lentivirus particles, which express caveolin-1-shRNA or a negative control shRNA, were used to infect RPMVEC (Targeting caveolin-1 shRAN sequence:GACGTGGTCAAGATTGACTTT;negative control sequence:GCTTTGTGATTCAATCTGTAA) [22]. 1×10^6^ RPMVEC were seeded in a 6 cm plate. The next day, the medium was replaced with fresh DMEM containing 15% fetal calf serum, lentivirus and the medium with 8 mg/ml polybrene. The supernatant with the lentivirus particles was replaced the following morning with media containing 15% fetal calf serum.

### Measurement of Transendothelial Resistance (TER)

An epithelial volt-ohm meter and STX-2 electrodes (EVOM; World Precision Instruments, Sarasota, FL, USA) was used to measure transendothelial resistance (TER, cm^2^) across the endothelial monolayer according to the manufacture’s protocol as described previously [19], [20]. RPMVEC (2×10^5^ cells/cm^2^) were seeded on gelatin-coated transwell polyester membranes and grown to confluence for 48 h. Cells were then allowed to stabilize for 24 h, incubated with fresh culture medium in the presence or absence of TNF-α for different time points. The changes of TER across RPMVEC monolayer were recorded.

### Measurement of Fluorescein Isothiocyanate (FITC)-BSA Flux Across Monolayers of Cultured Endothelial Cells

Endothelial cells were seeded on top of the transwell chamber in 24-well plates (0.4 µm pore size) and grown to confluence. The monolayers were serum-starved for 1 h then incubated with fresh culture medium in the presence or absence of TNF-α for the different time points, as described below. At the end of the stimulation, FITC-BSA (10 mg/ml, Sigma) and an equimolar amount of unlabeled BSA were mixed with phenol red-free DMEM and added to the top chamber and the bottom chamber, respectively, for 1 h at 37°C. Paracellular flux was assessed by obtaining aliquots from both chambers to measure the real-time changes of permeability across the endothelial cell monolayers. The concentration of FITC-BSA was quantified with a fluorescence spectrofluorophotometer as described previously [23]. The BSA flux was calculated as a ratio between the fluorescence intensity in the lower compartment and the upper compartment. The data were expressed as a percentage of the control.

### Fluorescent Staining

5×10^4^ RPMVECs were seeded on 2 cm^2^ coverslips and grown for 12 hours. The coverslips were washed with phosphate buffer saline (PBS) after drug treatment and then fixed with 4% formaldehyde. After blocking with 2% BSA for 1 h, cells were exposed to the primary antibodies overnight at 4°C. After washing with PBS, cells were incubated with the appropriate secondary antibodies conjugated to immunofluorescent dyes for 60 min. In order to stain for F-actin, fixed cells were incubated with rhodamine-conjugated phalloidin for 1 hour at 37°C,then washed with PBS. Coverslips were mounted on glass slides with 60% glycerol in PBS. Cells were scanned with the laser scanning confocal microscope.

### Rac1 Activation Assay

Determination of Rac1 activation (Rac1-GTP) was performed with a commercially available kit (Upstate, Lake Placid, NY, USA). Briefly, after stimulation, cell lysates were collected, and GTP-bound Rac1 was captured using a pull-down assay with immobilized PAK1-PBD according to the manufacturer’s protocols. The levels of activated small GTPases and total Rac1 were assessed by Western blot analysis and quantified by scanning densitometry of autoradiography films. The levels of activated proteins Rac1 were normalized to total Rac1 levels.

### Immunoblotting

Confluent monolayers were serum-starved overnight before treated with desired agents. The cells were lysed in RIPA buffer at 4°C. The lysates were centrifuged at 14,000×g for 15 min followed by heating at 95–100°C for 5 min. Equal amounts of protein from each sample were electrophoresed by 12% gels and transferred to 0.45 µm nitrocellulose membranes. The membranes were blocked with 5% nonfat milk for 1 hour at room temperature, then probed with different antibodies overnight at 4°C The blots were washed with wash buffer, followed by incubation with HRP-conjugated secondary antibody for 1 h at RT. The blots were visualized using an enhanced chemiluminescence kit. Blots were scanned and quantitatively analyzed by ImageQuant software.

### Statistical Analysis

Values were shown as the mean ± SD. Data were analyzed using a standard Student’s *t*-test or a one-way ANOVA. Significance in all cases was defined at *P*<0.05.

## Results

### TNF-α-induced Hyperpermeability of Primary RPMVECs Monolayer was Blocked by Activation of Rac1

**Figure 1:**
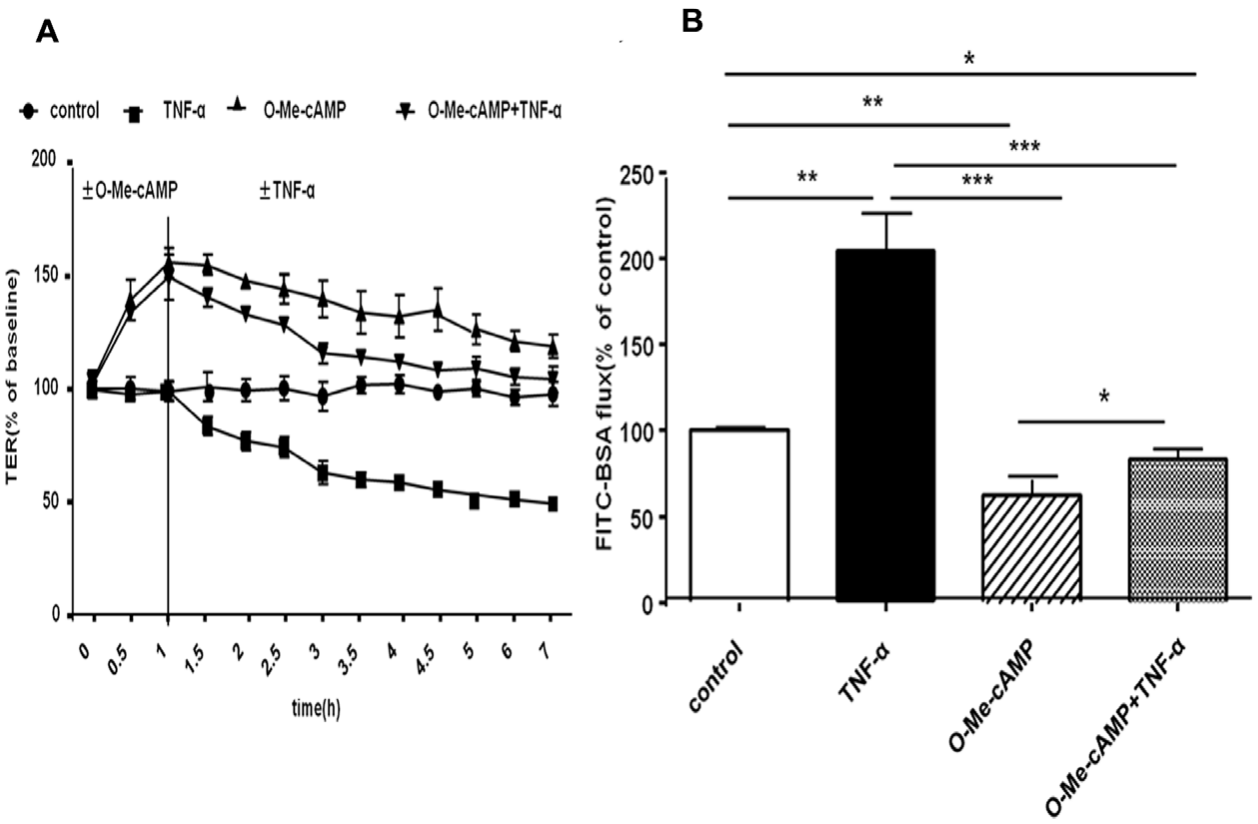
Effect of increased Rac1 activity on TNF-α-induced hyperpermeability of the primary RPMVEC monolayer. A: Effect on TER of the primary RPMVEC monolayer. Compared with controls, TER of primary RPMVEC monolayer challenged with TNF-α for 2 hours decreased significantly. Pretreatment of the primary RPMVEC with O-Me-cAMP (1 hour) significantly augmented TER and prevented TNF-α-induced the drop of TER. B: Effect on flux of FITC-BSA across the primary RPMVEC monolayer. Compared with untreated cells, the RPMVECs treated with TNF-α for 2 h had higher levels of FITC-BSA flux, whereas O-Me-cAMP treatment alone resulted in decrease FITC-BSA flux. Co-treatment with O-Me-cAMP and TNF-α [i.e., O-Me-cAMP +TNF-α] did not lead to increased endothelial permeability. Each bar represents mean ±SD of four independent trials; * denote *P*<0.05, ** denote *P*<0.01, *** denote *P*<0.001.

Initially, we determined the influence of TNF-α on EC barrier function. Primary RPMVECs monolayers were challenged with TNF-α (100 ng/ml) [24] or O-Me-cAMP (200 µM) [17], or O-Me-cAMP combined TNF-α stimulation. The changes in electrical resistance (TER) and FITC-BSA flux across confluent RPMVECs monolayers were monitored over time. Mean baseline resistance was 45.3±3.5 Ω*cm^2^. Exposure to TNF-α (100 ng/ml) for 1 hour, TER decreased significantly and then dropped to 63±5% of baseline levels after 2 hours. Negative controls showed no alteration of TER (Figure 1A). Pre-incubation with O-Me-cAMP alone, the agonist of Rac1, significantly augmented TER, which increased to 150±10% of baseline (Figure 1A) after 1 hour and resulted in a constant increased TER value for 6 hours. When pretreated with O-Me-cAMP for 1 hour and then administrated of TNF-α for 2 hours, TER remained increased and finally dropped to baseline levels after 6 hours. The flux of FITC-BSA was significantly increased to 205±23% of controls after 2 hours of TNF-α incubation (Figure 1B). Pre-incubation of endothelial monolayers with O-Me-cAMP for 1 hour significantly decreased the flux of FITC-BSA to 62±9% of controls. The application of TNF-α for 2 hours induced a decrease in the flux of FITC-BSA to 84±7% of controls (Figure 1B).

### Activation of Rac1 Led to TNF-α-induced Cytoskeleton Rearrangement and Cortactin Distribution

**Figure 2:**
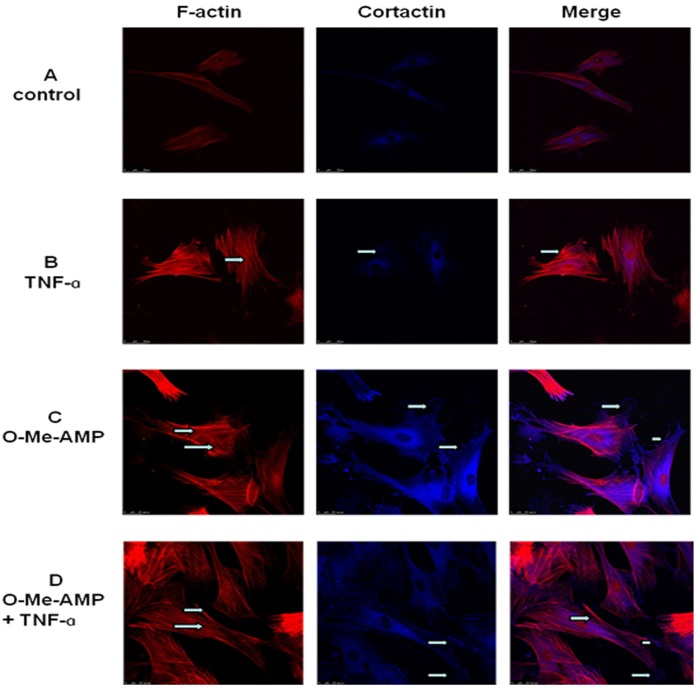
Effect of increased Rac1 activity on the rearrangement of F-actin and the distribution of cortactin. Cells were treated with TNF-α (B) for 2 hours or with O-Me-cAMP (C) for 1 hour or co-treated with O-Me-cAMP and TNF-α (D). In untreated cells, few stress fibers were seen, cortactin was detected both in the cytoplasm and along with some parts of the cell membrane (A). Exposure to TNF-α (B) induced stress fiber formation and the disappearance of cortactin from the cell periphery (shown by arrows); O-Me-cAMP (C) led to a loss of stress fibers and strengthened the peripheral actin band, moreover, caused an increase in the formation of lamellipodia and a change in cortactin localization to cell periphery forming a continuous linear staining pattern (C) (shown by arrows). Co-treatment with O-Me-cAMP prevented the TNF-α response (D) remarkably. Moreover, O-Me-cAMP induced lamellipodia increasing, dramatically attenuated the disappearance of cortactin from the cell periphery, and resulted in partial disappearance of central stress fibers (shown by arrows). All the images shown are representative of four independent experiments.

Immunoﬂuorescent images were generated in order to link the influence of TNF-α on EC TER with cytoskeleton changes. In the negative controls, few stress fibers were observed and cortactin was detected in the cytoplasm as well as along with some parts of the cell membrane (Fig. 2A). Primary RPMVECs exposed to TNF-α for 2 hours demonstrated a dramatic cytoskeleton rearrangement, which was characterized by the formation of central actin stress fibers and the disappearance of cortactin from the cell periphery (Fig. 2B). These changes were associated with the hyperpermeablilty of the endothelial cell monolayer. In contrast, O-Me-cAMP reduced the number of central stress fibers, enhanced the peripheral F-actin and caused an increase in the formation of lamellipodia. Moreover, O-Me-cAMP treatment led to recruitment of cortactin to the cell periphery resulting in the forming a continuous linear staining pattern (Fig. 2C). Pre-incubation with O-Me-cAMP for 1 hour, followed by the application of TNF-α for 2 hours led to a remarkable induction of lamellipodia, increased peripheral F-actin staining and dramatically attenuated the disappearance of cortactin from the cell periphery. Pre-incubation with O-Me-cAMP also resulted in partial disappearance of central stress fibers (Fig. 2D).

### TNF-α Dramatically Decreased Rac1 Activity in Primary RPMVECs

**Figure 3:**
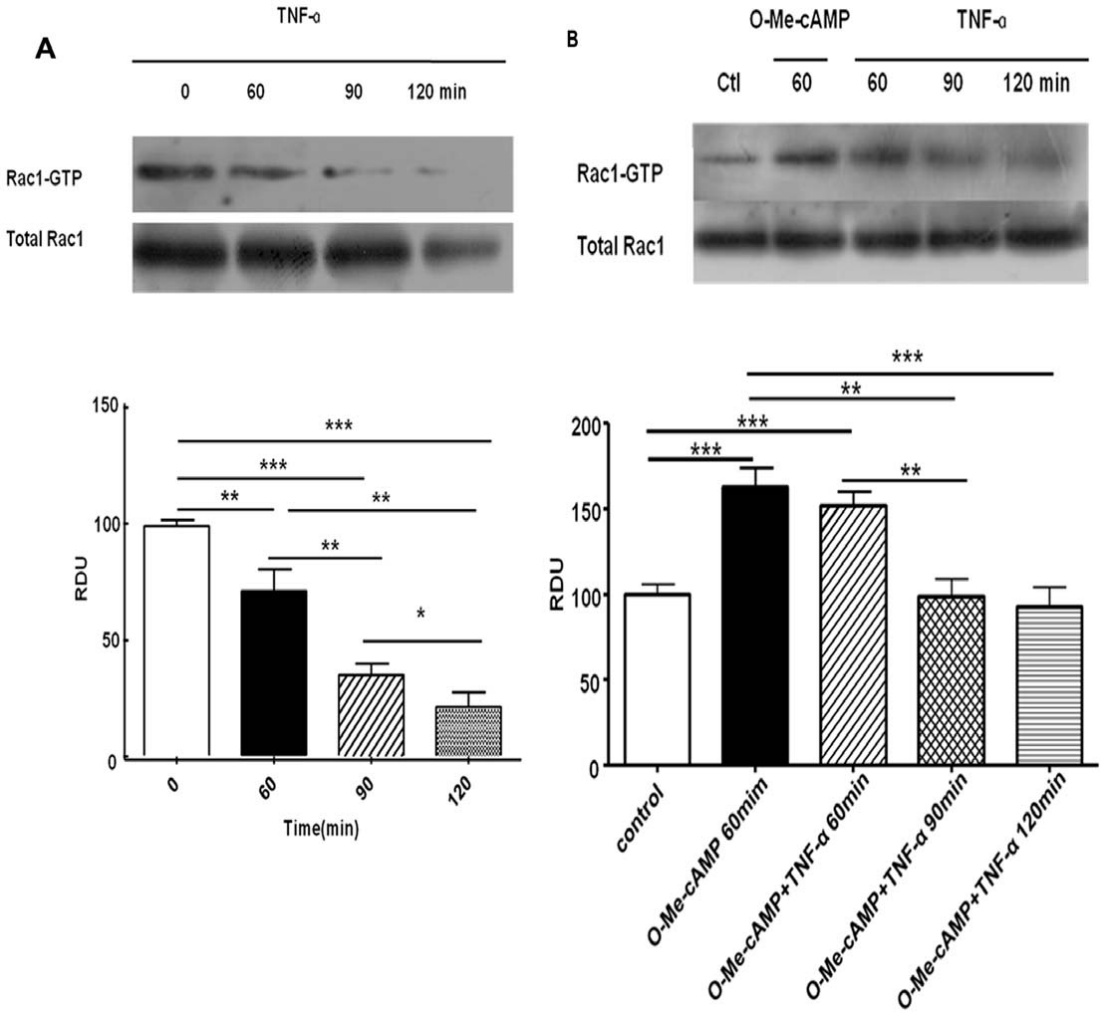
Effect of TNF-α, O-Me-cAMP or TNF-α combined with O-Me-cAMP on activity of Rac1. EC were stimulated with TNF-α(100 ng/ml) or O-Me-cAMP (200 mM) or TNF-α combined O-Me-cAMP for indicated time points. A: Effects of TNF-α on activity of Rac1 were evaluated using GTPase pulldown assays and normalized to the total GTPase content in cell lysates. After 60 minutes of TNF-α treatment, Rac 1 activity (n = 6) was significantly reduced to 71±10%, after 90 minutes, Rac1 activity was reduced to 35±5%(n = 6) and then more reduced to 21±6% (n = 6) after 120 minutes. B: O-Me-cAMP treatment alone (n = 6) resulted in significant activation of Rac1 to 163±11%. Pre-incubation of endothelial monolayer with O-Me-cAMP for 60 minutes before TNF-α treatment blocked Rac1 inactivation. Values are mean±SD. *P<0.05, **P<0.01, ***P<0.001.

Previous studies demonstrated that the activity of Rac1 was decreased in response to TNF-α and the activation of Rac1 could alleviate the loss of barrier function of microvascular endothelial cell, which was caused by cytokines [24]. However, the effects of TNF-α on primary RPMVECs are still unclear. Thus, we examined the activity of Rac1 in the presence of TNF-α with or without O-Me-cAMP in primary RPMVECs. As shown in Figure 3A, upon exposure to 100 ng/ml of TNF-α for 60, 90 and 120 minutes, the activity of Rac1 decreased depending on the different time points. After 60 minutes of TNF-α treatment, Rac1 activity (n = 6) was significantly reduced to 71±10% of controls. After 90 minutes, Rac1 activity was reduced to 35±5% (n = 6) and this activity was reduced to 21±6% (n = 6) after 120 minutes. O-Me-cAMP treatment alone (n = 6) resulted in a significant increase in Rac1 activation to 163±11% of controls. Pre-incubation of endothelial monolayer with O-Me-cAMP for 60 minutes blocked the decrease of Rac1 activity induced by TNF-α (Figure 3B).

### Down-regulation of Caveolin-1 Attenuated TNF-α-induced Breakdown of Monolayer Integrity in Primary RPMVECs

**Figure 4:**
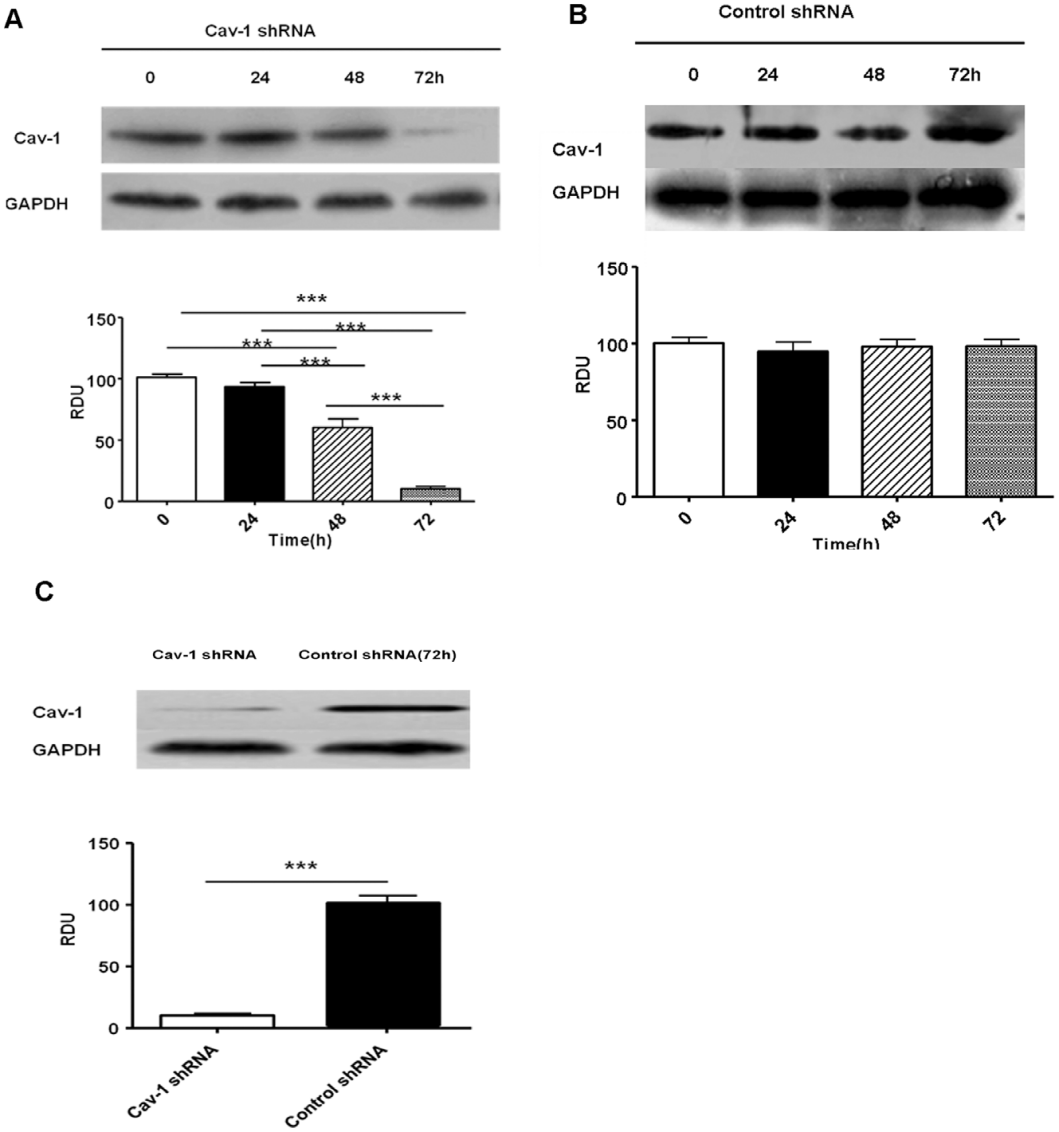
shRNA-mediated down-regulation of caveolin-1 expression in primary PMVECs. Control shRNA and Cav-1-shRNA cells were harvested and analyzed in immunoblots probed with a caveolin-1 antibody or GAPDH antibody, as shown in Figure 4. A. Caveolin-1 shRNA reduced expression of caveolin-1 obviously at the time point of 48 h(60±10%) and to minimum at 72 h (10±2%).B. Nevertheless, there was no change of caveolin-1 expression in control shRNA cells. C. Cav-1- shRNA reduced caveolin-1 expression by approximately 90% as compared with control shRNA. Values are mean±SD. *P<0.05, **P<0.01, ***P<0.001.

To determine the role of caveolin-1 in TNF-α–induced hyperpermeability of primary RPMVECs monolayer, we utilized shRNA to specifically down-regulate caveolin-1 expression. As indicated in Fig. 4A, caveolin-1 shRNA reduced caveolin-1 expression to 60±10% at 48 h and to minimum of 10±2% after 72 h. No changes in expression of caveolin-1 were observed with the control shRNAs (Fig. 4B). As indicated in Fig. 4C, caveolin-1 shRNA reduced caveolin-1 expression by approximately 90% as compared with control shRNA.

**Figure 5:**
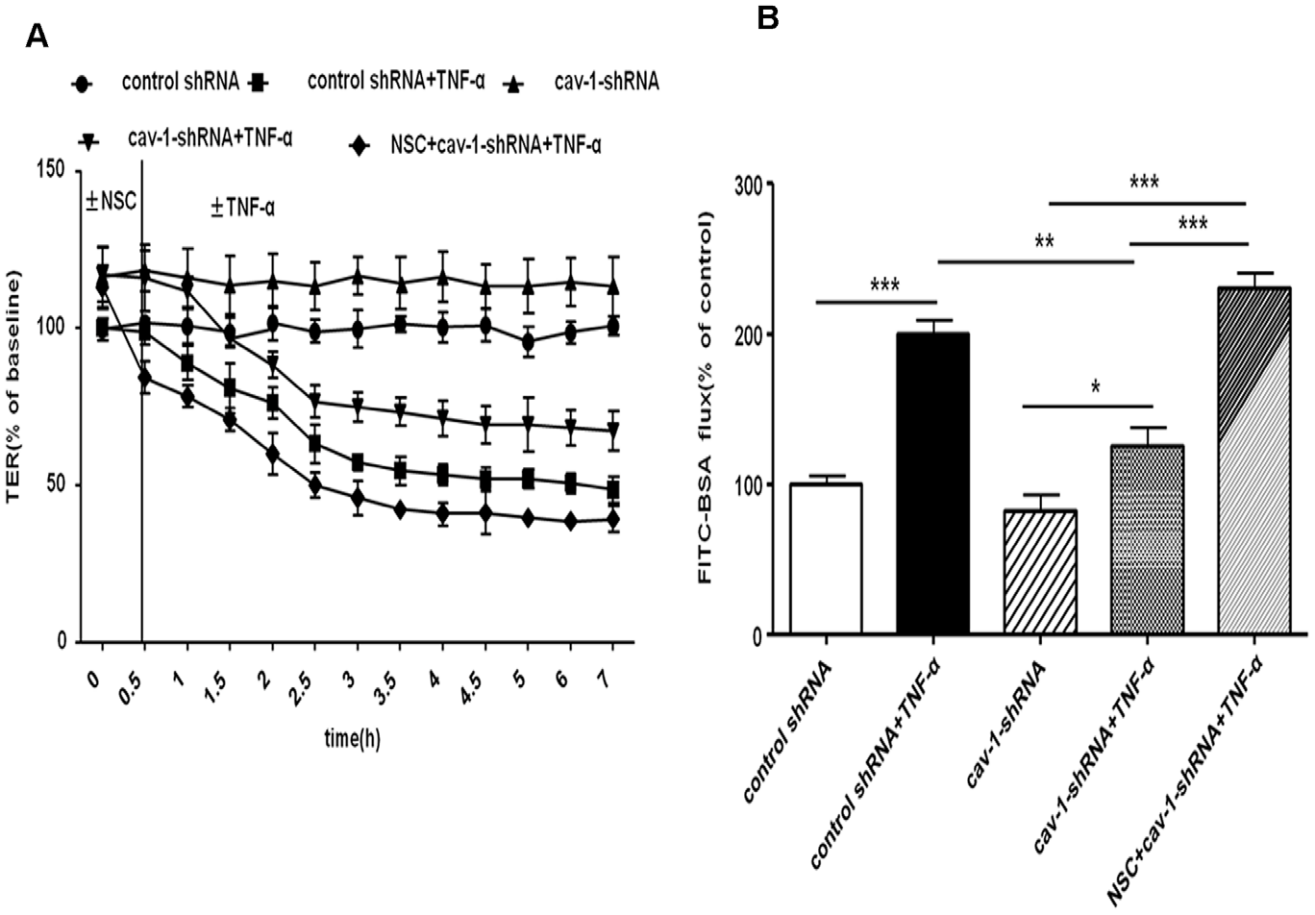
Effect of down-regulation of caveolin-1 on TNF-α-induced hyperpermeability of PMVEC monolayer. A: Effect on TER of PMVEC monolayer. Compared with control shRNA group, the mean baseline TER of PMVECs monolayer increased by 116±10% in Cav1-shRNA group. When challenged with TNF-α, TER of PMVECs monolayer decreased conspicuously in control shRNA more than in Cav-1-shRNA group. Compared with control shRNA cells at different time, prêtreatment with NSC-23766(30 min) significantly abolished the increasing TER in Cav-1-deficient cells monolayer with or without TNF-α stimulation. B: Effect on flux of BSA across PMVEC monolayer. The Flux of FITC-BSA in Cav-1-deficient cells monolayer decreased mildly. Treatment with TNF-α for 2 h significantly increased the flux of BSA in control shRNA group more than in cav-1-shRNA group. Moreover, co-treatment with NSC-23766 and TNF-α, the Flux of FITC-BSA of Cav-1-deficient PMVECs monolayer was increased more severely than that without application of NSC-23766. *P<0.05, **P<0.01, ***P<0.001.

**Figure 6:**
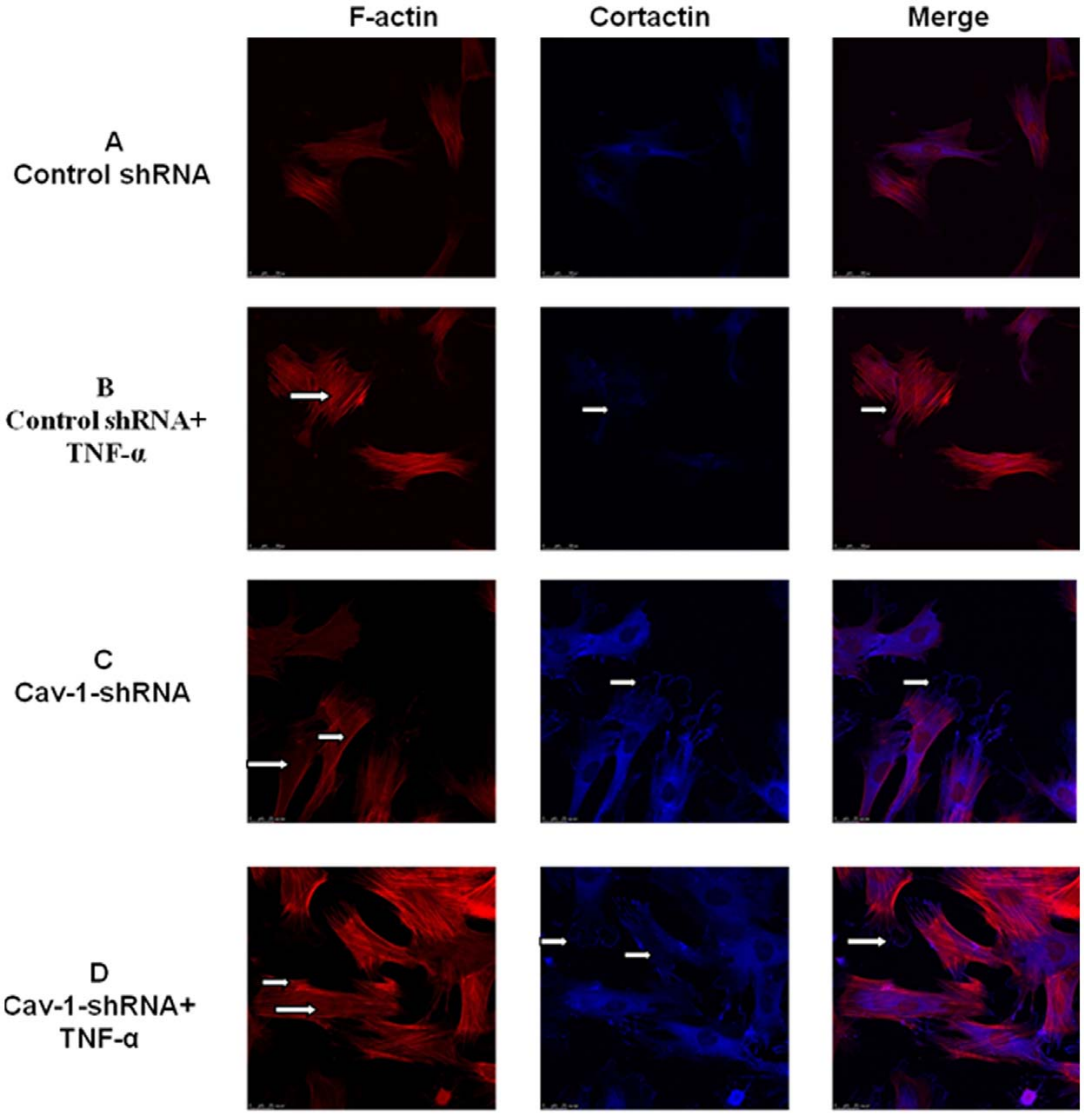
Effect of down-regulation of caveolin-1 on the rearrangement of F-actin and the distribution of cortactin. In control shRNA cells, few stress fibres were observed in the cytoplasm and cortactin was primarily located in the cytoplasm (A). Control shRNA cells exposure to TNF-α (B) induced stress fiber formation and the disappearance of cortactin from the cell periphery (shown by arrows); Down-regulation of caveolin-1 strengthened the peripheral actin band and induced an increase in the formation of lamellipodia and a change of cortactin localization to cell periphery forming a continuous linear staining pattern (shown by arrows) (C). Cav-1-shRNA cells exposure to TNF-α (D),remarkably, induced lamellipodia increasing and dramatically attenuated the disappearance of cortactin from the cell periphery, moreover, the levels of central actin stress fiber formation were not obvious (shown by arrows). All the images shown are representative of four independent experiments.

Down-regulation of caveolin-1 expression led to an increase in the mean baseline TER of RPMVECs monolayer to 57.8±4.9 Ω*cm^2^ by 116±10% compared with control shRNA cell monolayers (49.4±3.1Ω*cm^2^) (Fig. 5A). At the same time, the flux of FITC-BSA in Cav-1-deficient cells decreased (Fig. 5B). When Cav-1-deficient cells exposed to TNF-α, the TER decreased mildly and the amplitude of the decrease of TER was lower than that in control shRNA cell monolayers (Fig. 5A). Furthermore, the flux of FITC-BSA increased mildly, but the amplitude of the increased flux of FITC-BSA was lower than that in the control shRNA (Fig. 5B). These data indicate that the down-regulation of caveolin-1 can attenuate TNF-α-induced hyperpermeability of primary RPMVECs monolayers. Consistent with the changes of permeability in RPMVECs, down-regulation of caveolin-1 expression led to lamellipodia formation, cortactin translocation and increases in peripheral F-actin levels. Monolayers challenged with TNF-α led to obvious formation of lamellipodia, however, the disappearance of cortactin from the cell periphery was not obvious nor was a decrease in the levels of central actin stress fiber (Fig. 6).

### Down-regulation of Caveolin-1 Increased Rac1 Activity in RPMVECs

**Figure 7:**
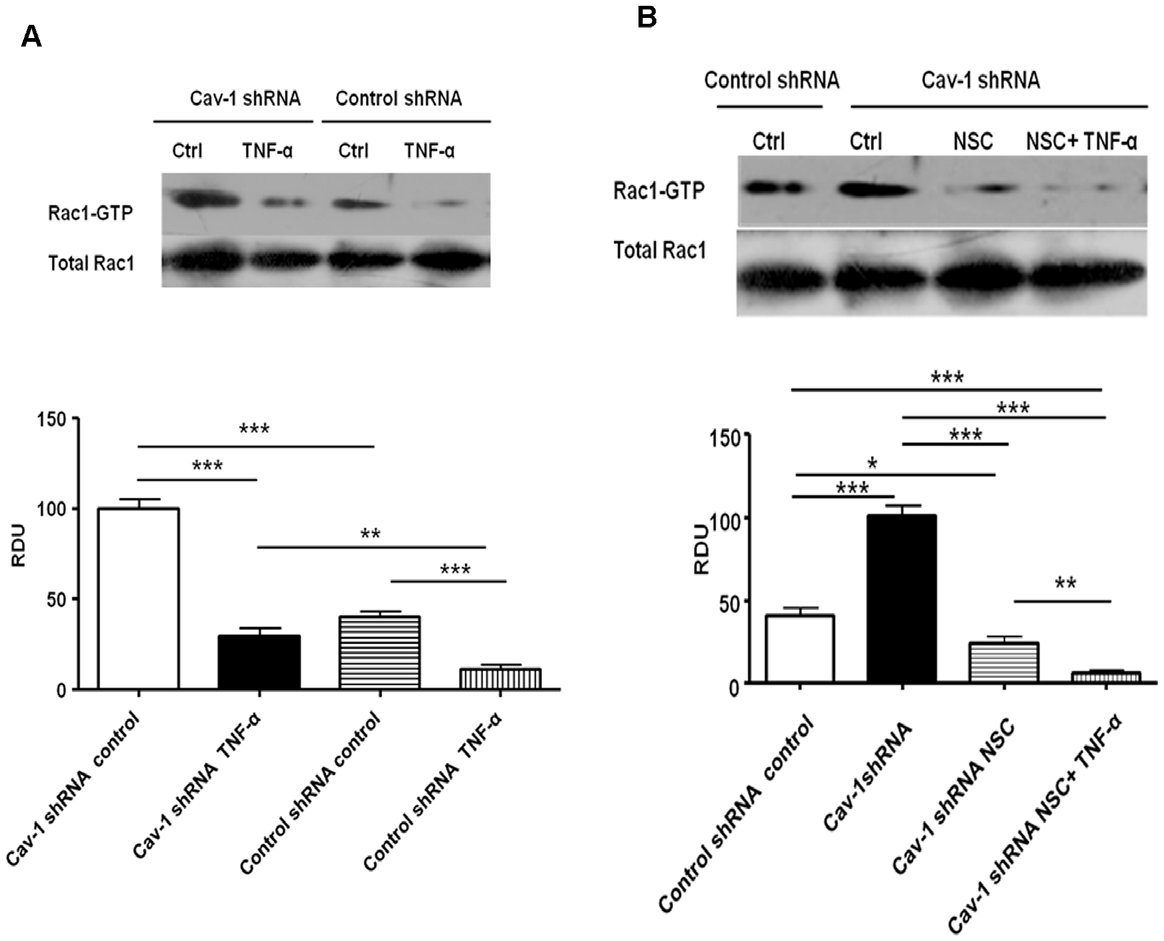
Down-regulation of caveolin-1 expression enhanced Rac1 activity, which was abolished by NSC-23766. A.Primary PMVECs transfected with control or caveolin-1 shRNA were challenged with TNF-α(100 ng/ml) for 2 hours. Rac1 activity in the cell lysates was evaluated using GTPase pull-down assays and normalized to the total GTPase content in cell lysates. Caveolin-1 deficient cells showed a significant increase in GTP-bound Rac1 compared with control shRNA cells in the resting state (2.5±0.2-fold increase, *P*>0.001) and after TNF-α stimulation (2.7±0.4-fold increase, *P>*0.01).B. Measurement of Rac1 activity by GTPase pulldown assays. Down-regulation of caveolin-1 expression resulted in increased activity of Rac1 to 250±20% of controls shRNA. Incubation with NSC-23766(200 mM, 30 min) abolished this effect. Co-treated with NSC-23766 and TNF-α, the activity of Rac1 decreased more than that treated with TNF-α alone. Each data point represents the mean±SD. derived from six independent experiments, *P<0.05, **P<0.01, ***P<0.001.

In our study, we observed that shRNA-mediated caveolin-1 loss enhanced Rac1 activity in the resting state and that the loss of caveolin-1 also attenuated decreased Rac1 activity induced by TNF-α stimulation in primary RPMVECs (Fig. 7A). Caveolin-1 deficient cells showed a significant increase in GTP-bound Rac1 compared with cells treated with the control shRNA (2.5±0.2-fold increase, *P*<0.001) and after TNF-α stimulation for 2 hours (2.7±0.4-fold increase, *P*<0.01) suggesting that caveolin-1 acts as a negative modulator of Rac1.

### Inhibition of Rac1 Activity Abolished the Barrier-protective Effect Induced by Down-regulation of Caveolin-1 in Response to TNF-α in RPMVECs

In the previous section, we have confirmed that inhibiting caveolin-1 could protect barrier function from injury of TNF-α in Cav-1-shRNA cells. We also observed that this barrier-protective effect induced by knocking down caveolin-1 was abolished by pharmacological Rac1 inhibitor NSC-23766 (200 mM, 30 min). The TER of Cav-1-deficient PMVECs monolayers were higher than that of the control shRNA cells,regardless of whether they were pretreated with TNF-α or not. Such TER increase could be inhibited by Rac1 inhibitor NSC-23766. Moreover,NSC-23766 could make the TER decrease more in PMVECs which losing caveolin-1 (Fig. 5A). Highpermeability to FITC-BSA of PMVECs could also be observed in this same process (Fig. 5B) Consisted with these changes,the elevated Rac1 activity in caveolin-1 low expressed cells was obviously suppressed by NSC-23766 (as shown in Fig. 7B). Therefore, we could conclude that inhibition of Rac1 activity abolished the barrier-protective effect induced by down-regulation of caveolin-1 in response to TNF-α in RPMVECs.

## Discussion

Inflammatory mediators induce vascular hyperpermeability primarily through the formation of intercellular gaps between endothelial cells, which is a process known as paracellular leak [25]. The mechanisms of gap formation can be widely contributed to three overlapping categories including [26]; i) injury and/or apoptosis of cells in the endothelial monolayer, ii) internalization or disassembly of intercellular junctions, and iii) remodeling of the actin cytoskeleton, leading to a change in cell shape. Rac1, the main GTPase, is known to regulate the cytoskeleton and intercellular junctions via cell spreading [27], buttress cortical actin rim formation [28] and reorganization of the junction-associated cortical actin cytoskeleton [29].

In the present study, we investigated the requirement of caveolin-1 in Rac1 signaling and the role of cortactin in the stabilization of endothelial barrier functions. We focused on cortactin because it is known to be important for endothelial barrier integrity and in previous studies endothelial cell barrier enhancement correlated with activation of cortactin [28]. On the other hand, Rac1-dependent translocation of cortactin to the cell periphery could be observed by confocal microscope in single cells so that we could assess endothelial function by changes in cell shape. We provide evidence that increased Rac1 activity leads to cortactin redistribution from the cytoplasm into membrane [30], which triggers peripheral actin polymerization [30] and formation of the peripheral actin rim, and thus EC barrier enhancement [17]. Moreover, our data demonstrate that Rac1 activity is negatively controlled by caveolin-1 and down-regulation of caveolin-1 can protect barrier function in the primary RPMVEC undergoing TNF-α stimulation.

Our experiments demonstrate that TNF-α induced barrier breakdown in cultured primary RPMVEC was a direct result of impaired Rac1 signaling. We observed an increase of central actin stress fibers and the disappearance of cortactin from the cell periphery when exposured to TNF-α. This cellular change was accompanied by decreased Rac1 activity. All these changes were associated with hyperpermeablilty of the endothelial cell monolayer. However, activation of Rac1 could enhance cortical actin rim formation resulting in the protection of endothelial barrier from injury due to TNF-α. In our study, activation of Rac1 induced lamellipodia formation [31], cortactin localization to the cell periphery and activated cell spreading, which is important to inhibit cell collapse and prevent gap formation [32]. Many reports have revealed that Sphingosine-1-phosphate (S1P) induced strong Rac1 activation and promoted the translocation of cortactin to the cell periphery and augmentation of the cortical actin ring. These events may account for its barrier-enhancing properties [33], [34]. Moreover, S1P signaling, via the Rac1 pathway [35] regulates the cortactin-Arp2/3 complex formation, which ultimately results in the formation of lamellipodia and endothelial spreading [36], [37].

Previous studies have shown that Rac1 associates with the scaffolding domain of Cav-1 through its hypervariable C-terminal domain and that Cav-1 is part of a negative-feedback loop that controls cell polarity, spreading and migration by regulating the degradation of activated Rac1 [38]. Cav-1-deficient cells lose normal cell polarity [39], [40], exhibit impaired wound healing [41], and have decreased Rho and increased Rac and Cdc42 GTPase activities [7]. We first observed that shRNA-mediated caveolin-1 knockdown enhanced Rac1 activity in the basal state and attenuated Rac1 activity decreasing induced by TNF-α in the primary RPMVECs (Fig. 7A). Cav-1 deficient cells showed a significant increase in GTP-bound Rac1 compared with control shRNA cells in the resting state (2.5±0.2-fold increase, *P<*0.001) and after TNF-α stimulation (2.7±0.4-fold increase, *P<*0.01), suggesting that caveolin-1 acts as a negative modulator of Rac1 in primary RPMVECs, which was consistent with other data [9]. These findings provide a biochemical rationale for our observations using confocal immunofluorescence microscopy (Fig. 6), in which Cav-1-deficient primary RPMVECs were associated with an increase in lamellipodia, translocation of cortactin to cell periphery and an enhancement of cortical actin distribution, all of which are cellular features characteristic of enhanced small GTPase Rac1 activity. However, when Cav-1-deficient cells were treated with TNF-α, the lamellipodia and translocation of cortactin remained obvious, nevertheless, the formation of central stress fibers were not obvious. All these changes were in contrast to control shRNA cells. These phenotypes suggested that down-regulation of caveolin-1 can attenuate the decreasing of Rac1 activity and the changing of cells shape induced by TNF-α. Moreover, to investigate whether these variations of Rac1 activity and cell shape correlated with changes in endothelial barrier functions in primary RPMVECs, we measured the ﬂux of FITC-BSA across monolayers of control shRNA cells and Cav-1-deficient cells. Under resting conditions, the permeability of FITC-BSA in Cav-1-deficient cell monolayers was slightly decreased compared with control shRNA cell monolayers, but not significantly different (Fig. 5B). However, when challenged with TNF-α, the permeability of control shRNA cells monolayers significantly increased, whereas the permeability of Cav-1-deficient cells mildly increased. Otherwise, the elevation of Rac1 activity in Cav-1-deficient cells could be suppressed by NSC-23766(specific Rac1 inhibitor).Permeability was increased compared with control shRNA cells monolayer in response to TNF-α. These results indicated that the mechanisms by which TNF-α increased permeability involved caveolin-1 and likely implied a reduction of Rac1 activity and Rac1-depended translocation of cortactin followed by endothelial barrier impairment.

**Figure 8:**
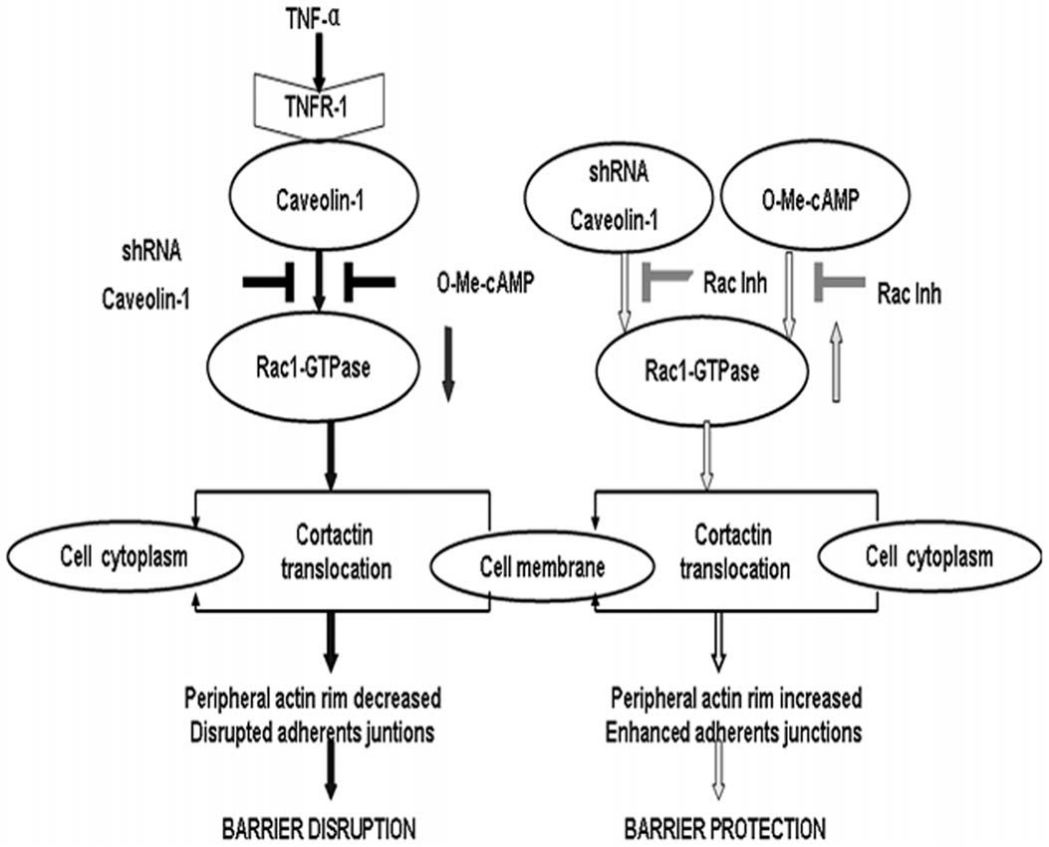
Proposed mechanism for caveolin-1-mediated modulation of endothelial barrier dysfunction induced by TNF-α. TNF-α induced the impairment of Rac1 signaling via TNFR-1 which locat in caveolae. This effect was further prevented by O-Me-cAMP. Finally, targeted knockdown of caveolin-1 activated Rac1 signaling that resulted in translocation of cortactin from cell cytoplasm to cell membrane, which promote enhancement of adherens junctions and peripheral actin rim and thus increase EC monolayer barrier properties. Therefore, knockdown of caveolin-1 completely abolishes TNF-α-induced barrier dysfunction, indicating that caveolin-1 plays a mechanistic role in TNF-α-induced endothelial cell activation.

Mechanistically, we have found that alterations in the signaling of Rac1 and cortactin are responsible for the changes in morphology and permeability in Cav-1-deficient cells. Previous reports have identified that down-regulation of caveolin-1 increases Src activation, which activates Rac1 through the exchange factors Dock180 [42], Tiam1, Vav2 [43], and FRG (through Cdc42 and Vav2) [44]. Furthermore, Cav-1 regulates polyubiquitylation and the consequent degradation of Rac1, which might increase Rac1 protein levels upon loss of Cav-1 expression [38]. Alternatively, the first step in the TNF-α-induced barrier breakdown of PMVEC monolayer is the binding of the cytokine to its receptor. It is reported that caveolae enriches tumor necrosis factor type 1 [45] and caveolin-1 form a complex with the TNF receptor [46]. Tumor necrosis factor receptor type 1 (TNFR-1) contains a death domain, which is required for TNF-α-induced proinflammatory cellular responses, for example, activation of NF-κB [47]. Furthermore, several studies have reported that silencing caveolin-1 could block TNF-α-induced proinflammatory responses [48], [49].More recently, We are awake that caveolins, especially caveolin-1, can bind to many types of plasma membrane receptor proteins and can concentrate these molecules within the caveolae [50] and further activates downstream signaling pathways. Therefore, we presume that the down-regulation of caveolin-1 prevents TNF-α from binding the TNFR-1 and influences the signaling transduction pathway, which results in partially preventing impairment of Rac1 signaling. Based on previous reports and the results of this study, we propose a new scheme that caveolin-1 regulates Rac1 activation and rat pulmonary microvascular endothelial hyperpermeability induced by TNF-α (Fig. 8).

However, it has to be emphasized that additional mechanisms, such as oxidative stress, may also contribute to the TNF-α-induced breakdown of endothelial barrier functions [51]. TNF-α can induce an intracellular oxidant stress via generation of Reactive oxygen species (ROS) [52].Reactive oxygen and nitrogen species (NO) are two major effector systems that are frequently implicated in the oxidative stress. It has been established that the enhanced production of reactive oxygen species (ROS) and diminished bioavailability nitric oxide (NO) lead to the microvascular dysfunction and that restitution of the normal balance between ROS and NO will recover the vascular function [53], [54]. Endogenous generation of oxidants could impair endothelial cell resulting in disruption of the interendothelial adhesion junctions (IEJs), actomyosin contractions, gap formation, and an increase in endothelial permeability [51], [55]. However, NO production could interact rapidly with superoxide and neutralize the oxidant production. NO donors or cGMP analogues can also reverse endothelial monolayer permeability induced by LPS or cytokines [56]. There is compelling evidence supporting that low levels of NO serve to stabilize endothelial barrier function and high levels serve to destabilize [57], [58].Under basal conditions, eNOS activation and function is inhibited by caveolin-1 because most of the intracellular pool of eNOS is associated with the scaffolding domain of caveolin-1 in endothelial cells [59]. Caveolin-1, as a negative regulator of eNOS activity, through regulating eNOS-derived NO production inhibits NF-κB activation and expression of proinflammatory proteins(iNOS and ICAM-1), when endothelial cells challenged by LPS or cytokines. Therefore, it has been believed that downregulation of caveolin-1 could result in increased NO production and a strengthening of the endothelial barrier [60].

In summary, our findings provided novel data demonstrating that TNF-α-induced RPMVECs barrier breakdown is, at least in part, mediated by Rac1 inactivation. Secondly, we confirm that down-regulation of caveolin-1 could attenuate permeability increasing induced by TNF-α partially by activation of Rac1-cortactin signaling pathway. Because the molecular basis of acute lung injury is not well understood and there are no specific therapies for it, this study may not only confirm the potential therapeutic value of caveolae in the management of ARDS but also depict a role of Rac1-cortactin signaling in maintainning RPMVECs barrier integrity. We believe that this research will deepen our recognition of lung vascular barrier function and may lead to novel treatments in ARDS.
